# The Chemical Composition and Biological Activities of Essential Oil from Korean Native Thyme Bak-Ri-Hyang (*Thymus quinquecostatus* Celak.)

**DOI:** 10.3390/molecules27134251

**Published:** 2022-07-01

**Authors:** Minju Kim, Kandhasamy Sowndhararajan, Songmun Kim

**Affiliations:** 1 School of Natural Resources and Environmental Science, Kangwon National University, Chuncheon 24341, Korea; scent@kangwon.ac.kr; 2 Department of Botany, Kongunadu Arts and Science College, Coimbatore 641029, India; sowndhar1982@gmail.com

**Keywords:** bak-ri-hyang, chemical composition, essential oil, biological activity, *Thymus quinquecostatus*, thyme

## Abstract

*Thymus quinquecostatus* Celak. (Korean name: bak-ri-hyang) is an important medicinal and aromatic herb in Korea, which is named for the spread of its fragrance over a distance of approximately 40 km. In traditional Korean systems of medicine, *T. quinquecostatus* has been used to treat cancer, constipation, hepatic disease, arteriosclerosis, poor circulation in women, constipation, and menstrual irregularities. At present, *T. quinquecostatus* is used only for ornamental and ground cover purposes. A literature search was conducted to retrieve information regarding the essential oil composition and biological properties of *T. quinquecostatus* from PubMed, Science Direct, Wiley, Springer, Taylor and Francis, Wiley, and other literature databases. *T. quinquecostatus* can be divided into different chemotypes, such as γ-terpinene, thymol, phenol, carvacrol, and geraniol, according to the presence of major components in its essential oil. Further, the essential oil from *T. quinquecostatus* has been reported to possess various therapeutic properties such as antioxidant, antimicrobial, anticancer, anti-inflammatory, analgesic, sleep prolonging, soothing, skin protection and whitening, anti-aging, anti-obesity, and anti-acne properties. In conclusion, this review will be helpful for utilizing the *T. quinquecostatus* plant in different industries including food, pharmaceuticals, pesticides, perfumery, and cosmetics.

## 1. Introduction

Essential oils are hydrophobic concentrates containing volatile aromatic compounds extracted from plants. Approximately 200 kinds of industrially used essential oils are obtained from the flowers, leaves, roots, stems, bark, fruits, buds, and seeds of various aromatic plants. Several methods are employed to isolate volatile components from medicinal and aromatic plants including hydro-distillation, steam distillation, extraction, supercritical fluid extraction (SFE), organic solvent extraction (OSE), and microwave extraction (AE). Among them, the hydro-distillation and steam distillation extraction methods are the standardized methods to isolate essential oils. Essential oils contain a variety of aromatic components, mainly belonging to monoterpenes and sesquiterpenes due to the differences in their odor characteristics. Essential oils are used in numerous industries to develop perfumery, aromatherapy, cosmetics, food, medicinal, and household cleaning products [1].

Essential oils from aromatic plants mainly contain monoterpenes and sesquiterpenes and their oxygenated derivatives with diverse chemical structures. These aroma components have been reported to possess various biological activities including anti-inflammatory, antispasmodic, analgesic, antioxidant, immunomodulatory, psychotropic, antiviral, antidiabetic, anticancer, and significant antibacterial properties [2,3]. Further, essential oils are applied in the fields of aromatherapy to maintain the physical and mental balance of humans. In agriculture, essential oils are used to inhibit the growth of plant fungal pathogens [4,5] and to control weeds [6,7]. Hence, essential oils can be used in eco-friendly agriculture in the future. In general, essential oils are widely used in the perfumery (fragrance) and the food industries (flavor) more than other industries. Although essential oils are produced from various aromatic plants in Korea, they are hardly used at the industrial level. 

The genus *Thymus* belongs to the family Lamiaceae with approximately 300 species distributed worldwide [8,9]. *Thymus* species have traditionally been used to treat digestive and respiratory diseases such as colds, indigestion, nausea, and dysentery [10]. Thyme species possess various pharmacological properties including anti-inflammatory, analgesic, antispasmodic, antitussive, carminative, antihypertensive, antidiabetic, and anthelmintic [11,12]. This plant has also been used to enhance the flavor of tea or spice in daily life. In particular, the main components in the essential oils of *Thymus*, thymol and carvacrol, are classified as Generally Recognized As Safe (GRAS) by the United States Food and Drug Administration (FDA). However, in the United States, the daily allowance of essential oils used in food is stipulated [13]. In *European Pharmacopoeia*, only the thymol chemotype is selectively used among thyme essential oils. Thymol (36.0–55.0%), carvacrol (1.0–4.0%), *p*-cymene (15.0–28.0%), γ-terpinene (5.0–10.0%), linalool (4.0–6.5%), β-myrcene (1.0–3.0%), and terpinen-4-ol (0.2–2.5%) are the most abundant components in the essential oils of *Thymus* species. Among them, thymol is considered in pharmacopeias and used as a food preservative in the US, UK, Japan, India, and China [10].

Thyme has been used in Korean oriental medicine for the treatment of upper respiratory tract inflammation. Research on thyme is only fragmentary and sporadic due to the fact of its sluggish industrial use. Hence, the cultivation of thyme is very limited on farms. In this review, the botanical and agronomic characteristics, the chemical composition of its essential oil, and the biological activities of Korean thyme (*Thymus quinquecostatus* Celakovsky) are summarized. This review paper provides sufficient knowledge to improve the industrial value of *T. quinquecostatus* and its cultivation practices on farms.

## 2. Botanical Characteristics

Thyme is a perennial deciduous shrub known to be native to the Mediterranean coast of southern Europe [14]. It is also native to Korea [15,16], China [17], Japan, Mongolia, Central Asia [18], and other subtropical and temperate regions. In the Lamiaceae family, the genus *Thymus* has a large number of species and is considered important among botanists because of its high commercial and medicinal value. *Thymus* is a Nepetoideae Kostel (subfamily), Mentheae Dumort (tribe) Menthinae Endl. (subtribe) [19], with 250 taxa in Europe, Northwest Africa, Ethiopia, Asia, and Greenland [20], and it is generally classified into 215 species [21]. Different species of *Thymus* have significant morphological variations and do not have clear diagnostic characteristics for plant characteristics. Hence, the total number of *Thymus* species varies greatly among researchers, making it the most controversial plant among those of European origin [22]. 

**Figure 1:**
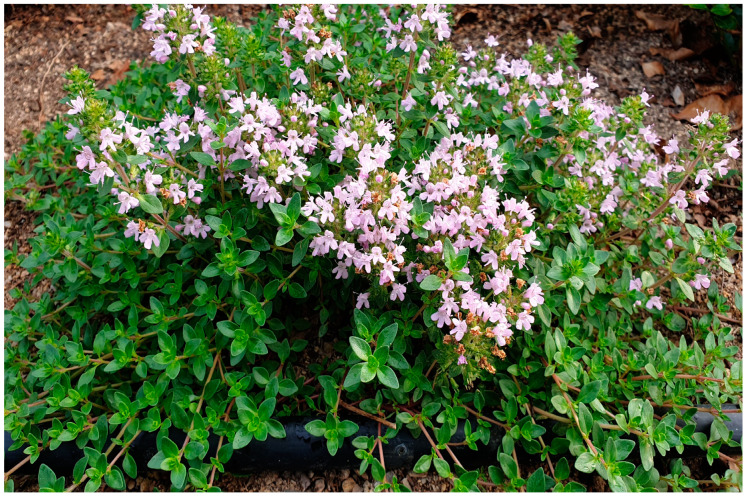
Fully grown *T. quinquecostatus* plant with flowers.

*T. quinquecostatus* has many branches from the stem and the leaves are opposite and egg-shaped, oval, or lanceolate (Figure 1). The length of the leaf is 5~12 mm and the width is 3~8 mm; the edge has almost no saw teeth, there are small hairs, and it has concave dots on both sides of the leaf. Thyme leaves have capillary secretory hairs, and Shin and Yu [23] observed this microstructure with an electron microscope, inferring that the oleaginous substance would be secreted by granular secretion. Jing et al. [24] also observed trichomes according to the development of thyme leaves and found that there are two types of peltate and capitate trichomes in thyme, and the components accumulated at each developmental stage of the leaf are different. These findings indicate that the chemical composition of thyme’s essential oils significantly vary according to the growing season.

The flowers of thyme are white or pink, 7–9 mm long, and bloom in July–August. The fruits are small, dark brown, and bear fruit in September [25]. Nakada and Sugawara [26] investigated the floral variation and reproduction mode of thyme. As for the stamen, there are long L-stamens and short S-stamens. It has been reported that anthers of type S-stamen produce pollen of reproductive fertility, whereas anthers of type S-stamen produce pollen of complete infertility.

In particular, two varieties of *T. quinquecostatus* are found in Korea: bak-ri-hyang (*T. quinquecostatus* Celakovsky), found throughout the Korean Peninsula; island thyme (T. *quinquecostatus* var. *japonica*), found only in island areas including Ulleungdo. Thymol was found to be the most abundant component in the essential oils of bak-ri-hyang (39.8%) and island thyme (54.7%). [27,28]. *T. vulgaris* L. is reported to be a completely different species from Korean native thyme. *T. quinquecostatus* is also distributed in China, but significant variations were observed in their essential oil compositions. Carvacrol (28.54%) is the major component of thyme from the Ningxia Guyuan region, whereas *p*-cymene (17.39%) is from the Shandong Yimeng region. These data suggest that the main component in the essential oil of thyme remarkably varied according to the region. The difference in the essential oil composition might be due to the differences in environmental conditions, harvesting time, cross-pollination, extraction, and analysis methods of the essential oils [29,30]. There is a great morphological difference between thyme. 

Currently, it has been investigated that several types of thyme introduced from foreign countries are being sold as domestic thyme in the private sector in Korea. Further, the correct identification of domestic thyme is too difficult due to the presence of many crossbreeds between native and imported thymes [31]. Therefore, a clear species characterization study of Korean native thyme is required. These studies will support the industrial needs following the entry into force of the Nagoya Protocol.

## 3. Agronomic Characteristics

Since thyme is an aromatic plant, the standard of cultivation in industrial terms is high yield and high quality of natural essential oil. The soil, light, planting distance, and cultivation methods for cultivating thyme containing high-content and high-quality essential oil have been studied. *T. quinquecostatus* can be propagated by seeds, but generally, cuttings are used. This takes advantage of the botanical characteristics of thyme stems that take root in the soil when they grow to a certain extent, but it is not possible to cut them at any time of the year. Choi et al. [32] reported that more than 80% of rooting was achieved when thyme was cultivated between April and June. After 30 days, the seedling qualities, such as rooting, plant length, leaf length, leaf width, and the number of branches, were investigated. The authors found that the appropriate cutting time for thyme propagation was between April and June. Farmers use plant growth regulators when making cuttings (personal communication with Kim Hee-yeon), but there has been no study on the effect obtained by using them. Choi et al. [32] reported that the rooting rate was the highest (93%) under a perlite:vermiculite ratio of 1:1 (*v*/*v*). This ratio is suitable for the propagation of thyme cuttings for improving the plant length and width of seedling quality. Sunlight, along with moisture, has a great influence on plant growth. When thyme was grown without shading, the fresh weight was high, and the flowering period was early. Conversely, due to the low root depth of thyme, if periodic irrigation is not performed, it will suffer from drying out. The planting distance affects the overall yield. In general, it is known that an increase in plant spacing is found to increase the yield of the plant. A similar pattern was observed in the case of baechohyang, which is grown as a medicinal plant on domestic farms. As the planting distance of thyme was farther, the fresh weight and dry weight of the plant increased. When the planting distance was 15 × 15 cm, the plant height of thyme was 27.8 cm, and the fresh weight was 48.7 g/plant. However, the planting distance was doubled, and when it was 30 × 30 cm, the plant height was 25.3 cm, and the fresh weight was 156.7 g/plant. From these results, it can be seen that the greater the distance of thyme planting, the higher the yield. The essential oil content was 3.4% in non-forestry cultivation, whereas the oil content was as low as 2.6% in open-field cultivation, which indicates that stable moisture supply and protection from damage caused by pests were effective [32]. 

## 4. Chemical Composition of Essential Oils

The essential oils from *Thymus* sp. Have long been used for medicinal purposes, perfumery, and in food industries due to the presence of various bioactive components and their unique aroma. Hence, thyme has been of interest to many academic and industrial researchers. The essential oil isolation from *T. quinquecostatus* and its major components are presented in Table 1.

A total of 30 volatile compounds were detected in the steam distilled oil of thyme using the solid-phase microextraction (SPME) technique. The main components were trans-geraniol (36.85%), citral (15.64%), 3-octanone (3.70%), geranyl acetate (3.43%), borneol (2.48%), and nerol (2.25%) [33]. A study indicated that 29 different compounds were detected in the essential oil of thyme extracted by steam distillation. Trans-geraniol (39.75%) was the main components followed by citral (20.04%), geranyl acetate (6.00%), borneol (3.22%), nerol (3.21%), and 3-octanone (2.49%). In China, the main component of thyme from Cheongwon is trans-geraniol, but the main component of thyme from Shandong Yimeng is *p*-cymene. The study suggested that the chemotype is different from that of Cheongwon and Shandong Yimeng of China [29]. It is reported that the different chemical types of thyme grown in different regions are due to the differences in genotype and environmental conditions [8,31]. Choi et al. [34] reported that the yield of thyme essential oil was 2.62%, and this level of content is not low compared to essential oils of other herbal plants; therefore, it is useful in industrial extraction. It was reported that the essential oil yield of other herbal plants was gamguk: 2.0%; pear cloves: 0.6%; lavender: 3.49%; rosemary: 2.89%; chamomile: 1.16%. 

Oh et al. [35] reported that cymene was the most abundant (93.5%) in the essential oil of Jeju thyme. Cymene was also found in the form of *p*-cymen-3-ol (50.41%), *p*-cymen-2-ol (24.06%), and cymene (19.04%). The results demonstrated that the chemical type of Jeju thyme is closer to thyme from Shandong Yimeng in China than thyme from Cheongwon Herbalang in Chungbuk [33]. From these results, it can be seen that thyme inhabiting Korea has various chemical types.

Lee et al. [36] extracted essential oil from the leaves and stems of thyme to test the functionality of native Korean herbal plants using three methods: supercritical extraction (SFE), steam distillation (SDE), and microwave extraction (MAE). When the leaves were extracted by the SFE, SDE, and MAE methods, the essential oil yields were 5.77, 5.72, and 5.70%, respectively, and there was no significant difference. There was no difference between the SFE method and the SDE method in the comparative study between the extraction methods performed on baechohyang in the same study by the authors. The MAE extraction method, used by Lee et al. [36] for the extraction of essential oils, uses microwaves and has the advantage of selectively heating and extracting only the desired components in natural substances [37]. It is also widely used in the extraction of essential oils [38,39].

In a comparative study on the content of island thyme essential oil, Lee et al. [37] reported that the efficiency of extraction with the SFE method was 0.20 to 0.27%, and the efficiency of the water steam distillation method was 0.03%. The authors found that this low extraction efficiency was different depending on the sampling location and harvest time, and this difference was also reported in the essential extraction efficiency of baechohyang [1].

Choi et al. [34] conducted a study on the harvest time, cultivation type, and drying method for the production of high-quality essential oil. In thyme, the essential oil content started to increase before the flowering period and was the highest during the flowering period (2.62%, steam distillation method), and after that, the content decreased significantly. Similar results were reported for baechohyang [40], indicating that the harvest time is very important for a high yield of essential oil. In a study on the volatile fragrance component of thyme according to the domestic production area, Chiang et al. [41] reported that there was a difference in the main component of essential oil. When thyme collected from the high mountains of Jeju Island was analyzed with a thermal desorption gas chromatograph-mass spectrometer, a total of 50 volatile components were detected in the essential oil, and the most abundant component was γ-terpinene (14.95%). A total of 62 volatile components were detected in thyme essential oil collected from the mid-mountainous region of Jeju, and the main component was *p*-thymol (27.51%). In the thyme collected from Ulleungdo, 41 types of volatile components were detected in the essential oil, and the main component was phenol (13.48%). A total of 54 components were detected in the essential oil of thyme collected in Gapyeong, Gyeonggi-do, and the main component was carvacrol (18.25%).

The results of these studies can be summarized as follows. First, the content of thyme essential oil differed by region. Second, it is possible to classify the chemical type as the main component of the essential oil of thyme. Chiang et al. [41] reported that there are at least four chemical types (i.e., γ-terpinene, *p*-thymol, phenol, and carvacrol) for thyme. The fact that the main component, as well as the content of the essential oil, may differ by region, even in the same species of plant, was reported in a study of thyme (*T. vulgaris*). The essential oil content of *Thymus spathulafolia* was 3.74% [42], *Thymus capitatus* was 6.0% [43], and *Thymus linearis* was 11.2% [44]. The main component of thyme from Casola and Modena, Italy, was thymol [45], but the main component of thyme from Palmaria Island was carvacrol [46]. 

In addition, Tohidi et al. [47] extracted essential oils of the genus *Thymus* collected from various regions in Iran. Through cluster analysis, they could be divided into three groups: thymol, geraniol/linalool, and carvacrol. These results indicate that even the same plant (thyme) living in one country may have different main components depending on the habitation area, and this difference is inferred due to the severe morphological variation of plants of the genus *Thymus.* In the western and central parts of the Balkan Peninsula, 250 taxa were identified [48] and only 110 taxa from the Mediterranean [49]. Hence, thyme has caused many taxonomic controversies among plant species of European origin [22]. Paying attention to the fact that bak-ri-hyang and island thyme are different in appearance, Shin and Kim [50] investigated the chemical composition of the essential oils. Thyme contains volatile organic compounds: thymol (41.7%) > *γ*-terpinene (16.0%) > *p*-cymene (13.0%) > *β*-caryophyllene (4.7%) > carvacrol (4.0%) > *β*-bisabolene (2.7%) > *α*-terpinene (2.6%). On the other hand, island thyme contains thymol (39.8%) > *γ*-terpinene (9.9%) > *p*-cymene (5.5%) > camphor (5.0%) > *β*-caryophyllene (4.7%) > *α*-pinene (3.5%). However, the most abundant component of both plants was thymol; thus, both plants can be classified as the same chemical type.

In a study by Shin and Choi [51], Korean thyme, which inhabits the entire Korean Peninsula, was classified into Jeju-do, Ulleung-do, and Korean-style. Baik et al. [52] reported that the content of the extracted components was different when domestic thyme was extracted with different solvents. The content of thymol was 64.23% in methanol extraction, 72.65% in ethanol extraction, and 57.98% in hexane extraction, indicating that the extraction solvent affects the extraction of specific components.

**Figure 2:**
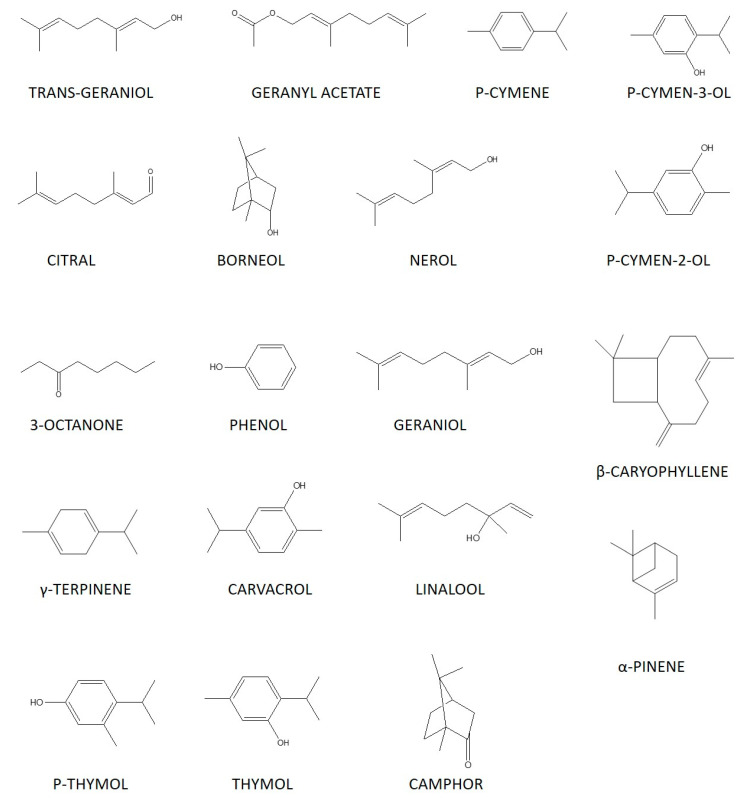
The chemical structure of certain important components in the essential oil of *T. quinquecostatus*.

The structure of the chemical substances contained in thyme essential oil, reported by many researchers to date, is shown in Figure 2.

## 5. Biological Properties

**Figure 3:**
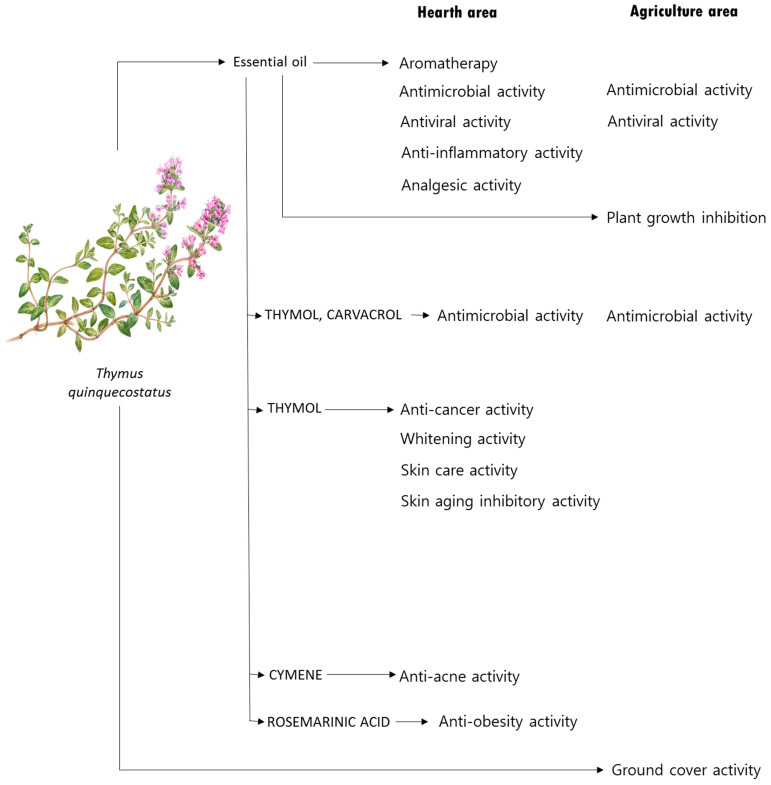
Biological activities of the phytochemicals found in the *T. quinquecostatus* plant. A miniature of the *T. quinquecostatus* plant was drawn by miniature specialist, Ms. Young Suk Lee.

*T. quinquecostatus* has various biological properties such as antioxidant, antimicrobial, anticancer, anti-inflammatory, analgesic, anti-obesity, and anti-acne activities. It has been reported to have various physiological activities, including sleep prolonging, soothing, whitening, skin protection, skin aging inhibition, and plant growth inhibitory activities. The biological activities reported so far are shown in Figure 3 and Table 2.

### 5.1. Antioxidant Activity

The US FDA defines antioxidants as substances used to preserve food by delaying deterioration, acid waste, or discoloration caused by oxidation. The oxidation reaction is one of the rancidity pathways of oils and fats. Oxidation products can be toxic when ingested as well as flavor in foods. Thus, the rancidity of oils and fats must be suppressed in terms of food preservation as well as stability. The rash is largely caused by a noncomplex diene compound produced by the reaction of singlet oxygen with unsaturated fatty acids by autoxidation by free radical chain reaction and enzymatic oxidation by lipoxygenase. All antioxidants act to block or delay their oxidation through several pathways [53]. Therefore, it can be said that the main function of antioxidants is the process of creating complexes by donating electrons or hydrogens to oils and fats containing free radicals. Representative natural sources of antioxidants identified so far include ascorbic acid, tocopherol, carotenoids, and flavonoids [53]. Thyme, clove, and other spices have been confirmed to have antioxidant effects [54].

The antioxidant effect of solvent extract of thyme has been reported by several researchers. Hyun et al. [55] reported that the ethyl acetate fraction from methanol extract of thyme has a high antioxidant effect, and flavonoids are effective compounds for radical scavenging. Kim et al. [56] reported that the methanol extract of thyme contained 41.37 mg/g of total flavonoids and that the main component of flavonoids was found to be quercetin (1.09 mg/g). The authors suggested that the antioxidant effect of thyme was high because the extract had a higher peroxyl radical scavenging activity than that of other Lamiaceae members. The α-glucosidase inhibitory potential of quercetin present in thyme was 80%, and the sucrase inhibitory activity was concentration dependent. Quercetin is a flavonoid that has been used for a long time and is known to possess antioxidant, anti-inflammatory, antiviral, anti-obesity, antidepression, anticancer, and antidiabetic activities and is useful against asthma and cardiovascular diseases [57].

Choi et al. [58] also reported that the extract of thyme had antioxidant effects after extracting seven kinds of domestic herbs, including thyme, and four kinds of imported herbs with ethanol and measuring their antioxidant activity. The essential oil of *Thymus lanceolatus*, which is related to thyme, contains 74.85% of oxygen-containing monoterpenes and 19.77% of monoterpene hydrocarbons, reported that it is similar to the antioxidant activity of thyme essential oils [59].

### 5.2. Antimicrobial Activity

Many researchers have reported on the antibacterial action of essential oils from different species of *Thymus*. Nazzaro et al. [60] explained the antibacterial action of essential oils of *Thymus* species on plant morphology due to the breakdown of the fat membrane, inhibition of the protoplast efflux pump, mitochondrial activity inhibition, production of oxygen oxides and nitrogen oxides, and the effects on mitotoxin production. Shin and Kim [50] studied the antibacterial activity of thyme essential oils against seven types of fungi (i.e., *Aspergillus niger*, *Aspergillus flavus*, *Blastoschyzomyces capitatus*, *Candida albicans*, *Candida utilis*, *Cryptococcus neoformans*, *Trichosporon mucoides*, and *Trichophyton rubrum*). *T. quinquecostatus* showed significant inhibition of the growth of the tested fungi with the minimum inhibitory concentration (MIC) in the range of 40–390 µg/mL. In addition, the well-known antibiotic, ketoconazole showed antifungal activity against these pathogens with MIC values between 3.12 and 12.5 µg/mL. In a follow-up study, Kim and Shin [61] investigated the inhibitory effect of essential oil of island thyme on *Candida albicans*, which causes inflammation in the vagina of Swiss albino female mice. The authors reported that the bacterial growth and the sterilization effect were very high. Thyme essential oil (4%) is expected to be used in the pharmaceutical industry in the future due to the fact of its effective antifungal potential. Further, *T. quinquecostatus* showed prominent synergistic effects with clotrimazole.

Park et al. [62] reported that thymol from thyme essential oil showed antimicrobial activity against several plant pathogens but was particularly effective against *Botrytis cinerea*, which produces disease in cucumbers. This study suggests that the oil can also be used in agriculture. Just as the essential oil of thyme exhibits antimicrobial activity, the extract of *Thymus laevigatus*, also exhibited antimicrobial activity [63]. It was reported that the antibacterial activity was high against *Aspergillus fumigatus*. The methanol extract contains flavonoids, tannins, anthraquinones, and phenolic compounds, and the dichloromethane extract contains volatile oils, mainly terpenoids. The authors reasoned that antibacterial action would be due to the presence of phenylpropanoids and tannins. Lihyang supercritical extract (SFE) showed antibacterial activity against *Ascosphaera apis*, which hardens and kills bees because spores invade through the mouth of bee larvae and the mycelium envelops the entire body [64]. When the thyme SFE is treated with a chalk pathogen, the mycelium takes on a light red band, and the growth of the mycelium is inhibited. Due to the presence of side effects, the experiment did not progress significantly. Since thymol acts like phenol, it is toxic even at low concentrations, and it is chronically toxic not only to microorganisms but also to insects (Maize weevil (*Sitophilus zeamais*)) [65].

Choi et al. [66] reported antimicrobial activity against various agricultural pathogens. The authors stated that thymol was reported to be effective against gray mold blight (*Botrytis cinerea*), leaf blight (*Rhizoctonia solani*), red pepper blight (*Phytophthora capsici*), red pepper anthrax (*Glomerella cingulata*), apple spot blight (*Alternaria mali*), and rice blast (*Magnaporthe grisea*). Thymol significantly inhibited the growth of these tested pathogens from 7.0% to 20.8% at a concertation of 100 µg/mL. 

Shin and Kim [50] reported the MICs for the pathogens of essential oils extracted from thyme and island thyme, and it was inferred that the bactericidal activity was due to the presence of thymol and carvacrol. Shin and Kim [67] confirmed the bactericidal effect of thyme essential oil on antibiotic-resistant strains through a follow-up study. The MIC for *Staphylococcus pneumoniae* was 0.125 to 1.000 mg/mL and the MIC for *Salmonella enteritidis* was 8 to >16 mg/mL. The thyme oils also exhibited significant antibacterial activity against *Staphylococcus aureus* and *Salmonella typhimurium* with MIC values in the range of 1 to 8 mg/mL. Further, the authors compared the antibacterial activity of essential oils with antibiotics such as norfloxacin (MIC at 2 to >64 µg/mL), oxacillin (MIC at 1 µg/mL to >16 µg/mL), and erythromycin (MIC at 0.25 µg/mL to >64 µg/mL). 

Thyme extract was also reported to have antibacterial activity. Choi et al. [58] reported that thyme extract inhibited the growth of *Escherichia coli* (approximately 50%), which is a contamination indicator and Gram-negative putrefactive bacterium. Products containing these essential oils are already on the market, and “DMC Base Natural”, a food preservative produced by DOMCA S. A. in Spain, contains 50% of essential oils of rosemary, sage, and citrus [68].

Previously, several authors reported that essential oils obtained from different species of *Thymus* exhibited considerable antimicrobial activity [69,70,71,72]. Yan et al. [73] stated that thymol, carvacrol, eugenol, geraniol, linalool, and nerol contained in thyme essential oils inhibit enzyme 1-deoxy-D-xylulose-5-phosphate reductoisomerase (DXR). DXR is an important enzyme that regulates the rate in the MEP terpenoid biosynthesis pathway, and the IC_50_ value of thyme essential oil for DXR enzyme is 48 μg/mL, showing a moderate inhibitory efficacy. Thus, the authors reported that carvacrol showed an excellent enzyme inhibitory ability even at a low concentration (68.3% at 20 μg/mL).

In thyme essential oil, carvacrol (5-isopropyl-2-methylphenol) is a monoterpene isomer of cymene, also known as 2-hydroxycymene. It has been reported that carvacrol disrupts the membrane of microorganisms to cause leakage of intracellular ions and lowers the proton motive force of cells by inhibiting ATPase activity [74]. In addition, *p*-cymene [75], cinnamaldehyde [76], cinnamic acid [77], and eugenol [78] are also known to disrupt cell membranes. It is concluded that thyme essential oil exhibits antimicrobial activity through the combined action of several monoterpene compounds.

The essential oil contained in thyme showed antimicrobial activity against food microorganisms, and Burt [68] reported that Gram-positive microorganisms were much more sensitive than Gram-negative microorganisms. It was demonstrated that the essential oil killed the microorganisms by dissolving the cell membrane and mitochondrial membrane and eluting the cell material. Since the essential oil of *T. laevigatus* also exhibited antimicrobial activity [63], it is inferred that antimicrobial activity is a common characteristic of plants of the genus *Thymus*, including *T. quinquecostatus*.

### 5.3. Antiviral Activity

Orchard and van Vuuren [79] reported that *T. vulgaris* had a CC_50_ value of 0.0079% for HSV-1/HSV-2, and an IC_50_ value of 0.0010/0.0007%, which was effective in treating skin diseases caused by viruses. *Thymus* essential oil is inferred to have antiviral activity, but scientific proof for this has not been made so far. Antiviral studies of thyme essential oil are expected to be widely applied in the treatment of various diseases.

### 5.4. Anti-Inflammatory Activity

In an anti-inflammatory study of thyme, Kim and Suk [80] found that intraperitoneal administration of essential oil from Ulleungdo (20, 40, and 80 mg/kg) showed a concentration-dependent analgesic effect. It was reported that when 80 mg/kg was administered, it was more effective for pain than the control drug, aspirin (70 mg/kg) administered group. The authors reported that ear edema was suppressed in a concentration-dependent manner when 60, 120, and 240 μg/ear of thyme essential oil was administered through the ear edema test in mice. It was concluded to be effective in anti-inflammatory activity. However, the anti-inflammatory mechanisms of thyme essential oil need to be discovered prior to clinical study.

### 5.5. Anticancer Activity

The anticancer effect of thymol, one of the main components contained in thyme, is well summarized in the literature by Islam et al. [81]. Thymol and its derivatives possess effective anticancer activity in different test systems such as mice, rats, and cancer cell lines. Thymol is effective against a variety of cancer cells, including glioblastoma cells, glioma cells, breast cancer cells, leukemia cells, mastocytoma cells, cervical cancer cells, laryngeal carcinoma cells, gastric carcinoma cells, and neuroblastoma cells [81,82]. The IC_50_ values of thymol varied significantly according to the type of cell line tested. The IC_50_ value of thymol for the breast cancer cell line MCF-7 is 304.81 µg/mL, while for leukemia cell lines, P388 is 0.8 µg/mL, hepatocellular carcinoma cell HEPG2 is 266 mM, and HL-60 is 113.51 µM. In the case of the cervical cancer cell, HeLa, the IC_50_ value of thymol is 134.29 µg/mL [82]. In particular, *T. vulgaris* essential oil showed antiproliferative and proapoptotic effects in MCF-7 and MDA-MB-231 breast cancer cells. Further, *T. vulgaris* essential oil at 0.1% and 1% doses reduced the volume of tumors in a syngeneic 4T1 mouse model and also decreased the tumor frequency by 53% compared to the control (at 1%) in the rat model [83]. Another study indicated that thymol isolated from *T. vulgaris* prevents colorectal cancer by inhibiting the Wnt/β-catenin pathway in HCT116 and Lovo cells as well as in BALB/c nude mice [84]. In a recent study, the essential oil of *T. vulgaris* registered the strongest antiproliferative activity against the MCF-7 cell line (IC_50_: 52.65 μg/mL) followed by the H460 (IC_50_: 68.59 μg/mL) and MOLT-4 cell line (IC_50_: 228.78 μg/mL) [85].

### 5.6. Analgesic Activity

Beer et al. [86] reported that thymol was effective for analgesia because thymol affected the spontaneous contractile activity of the smooth muscle of the stomach and vena cava of guinea pigs in an in vitro experiment.

In an experiment confirming the analgesic action of thyme essential oil on the central nervous system (CNS), analgesic effects were found at 20, 40, and 80 mg/kg in a hot plate test, and at 80 mg/kg, the analgesic effect was found to be more peripheral than the CNS. It showed a greater effect in the peripheral nervous system (PNS) [80]. However, research on whether the analgesic effect of thyme is also effective in humans has not yet been conducted.

### 5.7. Sleep Prolongation Activity

Hypnotics refers to drugs that make the user sleepy and promote the onset and maintenance of wavelengths similar to those of natural sleep. Bromide, known as a sedative in 1853, has been used as a sleeping pill since 1864. In addition, chloral hydrate, paraldehyde, urethane, etc., were used before 1900. A variety of barbiturates have been used, but their effects do not last for more than 2 weeks, and safety issues have been raised; thus, they are not currently used. Since chlordiazepoxide appeared in 1961, similar drugs have been produced and used as sleeping pills [87]. In a pentobarbital induced-sleep test in a mice model, *T. magnus* essential oil (at 100 mg/kg, i.p.) significantly increased the sleep time (222.9 min) when compared to the control group (70.1 min) [80]. 

### 5.8. Calming Activity

Kim and Suk [80] reported that thyme essential oil (100 mg/kg) was administered to mice, and no significant effect was found in the forced-swimming test and open-field test. It can be said that there was no evidence of a calming effect.

### 5.9. Whitening Activity

Whitening is classified as a functional cosmetic according to the Cosmetics Act. According to the Ministry of Food and Drug Safety, they are (1) cosmetics that prevent melanin pigment from being deposited on the skin to suppress the formation of spots or freckles and (2) the color of melanin pigment deposited on the skin. It is defined as a cosmetic with the function of lightening the skin by thinning the skin. It has been announced and recognized as a whitening ingredient [88]. In particular, tyrosinase enzyme is highly linked with melanin synthesis. Most of commercially available skin whitening products are tyrosinase inhibitors. The downregulation of tyrosinase gene expression is the most prominent approach for the development of whitening ingredients [89].

Choi et al. [90] reported that when thymol was treated in mouse-derived melanoma cells (B16F10 melanoma cells), more than 80% of cell viability was maintained up to 200 μM concentration as well as inhibiting the expression of tyrosinase by α-MSH stimulation. These results can be said to show that thymol inhibits tyrosinase activity to exhibit whitening activity. Lee et al. [91] reported that Melasolv^®^, a thymol ester complex (3,4,5-methoxycinnamate thymol ester), inhibits melanogenesis in melanin-α and α-MSH-stimulated B16 cells. As evidenced by microscopic observation, it was reported that the complex inhibits the migration of melanin to multiple epidermal layers. Summarizing the results of the studies so far, it can be said that thymol is an important functional material that causes whitening activity, although slightly toxic to humans.

### 5.10. Anti-Obesity Activity

A study demonstrated that thymol detected in the hexane solvent fraction of the extract had a high alpha-glucosidase inhibitory activity, and thus had an anti-obesity effect [55]. Lee et al. [92] also studied the activity of lipolytic enzymes by examining polyphenol components contained in the aboveground part of Ulleungdo-grown island thyme. Rosmarinic acid in thyme extract is expected to have an anti-obesity effect, and it will be utilized for the development of anti-obesity agents [93].

### 5.11. Skin Protection Activity

Ultraviolet rays (UV) that reach human skin from the sun are a major environmental factor that cause skin aging. In particular, polyphenol-rich compounds are known to prevent skin aging. Ultraviolet rays that reach human skin are mainly UVA (400–315 nm) and UVB (280–315 nm), but when overexposed, it causes retinal destruction of the eyes, erythema, inflammation, and serious consequences for the immune system as well as photoaging and keratinization. The development of cellular malignancies and melanomas has been reported in academia [94,95,96,97].

The effect of *T. vulgaris*, and the thymol contained therein, in a skin aging study was investigated. *T. vulgaris* extract (1.82 μg/mL) and thymol (1 μg/mL) were used to induce UV-induced skin damage (epidermis). It was shown to have a protective effect on cell morphology, proliferation, cytotoxicity, and genotoxicity [98]. Thyme also contains a higher level of thymol content; therefore, it is expected to have a UV protection effect and to be used in future cosmetic development.

### 5.12. Skin Aging Inhibitory Activity

UVB is known to induce oxidative stress in human skin and induce transient or persistent genetic damage, increase in activation factors, and expression of aging factors such as matrix metalloprotease (MMP). Exposure to UVB increases the expression of MMP protein, a type of collagenase that promotes skin aging and, as a result, collagen, a major protein constituting the skin dermis, is degraded or production is reduced [99]. Jung et al. [100] found that 70% ethanol extract of thyme strongly inhibited MMP-1 mRNA expression and protein expression by inducing phosphorylation of MAPK signaling factors such as ERK 1/2, JNK 1/2, and p38 kinase in human keratinocytes (HaCaTs). The study indicated that thyme extract was found to inhibit skin aging.

Another study reported that thymol from *T. vulgaris* extract showed an anti-aging effect [98]. Calo et al. [101] reported that both *T. vulgaris* extract and thymol inhibited the production of reactive oxygen species in keratinocytes cell lines treated with UVA/UVB. However, MDA formation was reduced only in UVA-treated cells, suggesting that *T. vulgaris* extract and thymol acted as antioxidants and scavengers of free radicals but did not directly reduce or prevent DNA damage. The anti-aging research results of thyme suggest that thyme essential oil can be applied as a good material for the development of anti-aging cosmetics.

### 5.13. Anti-Acne Activity

Acne is a chronic inflammatory skin disease that is caused by excessive sebum secretion, stress, hormone imbalance, misuse of cosmetics, and internal diseases. It is also called folliculitis because of irritation caused by the process of decomposing sebum and producing free fatty acids by breeding *Propionibacterium acnes* in hair follicles when pores are clogged due to the excessive sebum production [102].

Many researchers reported that essential oils are effective in treating acne such as tea tree (*Melaleuca alternifolia*) [103], eucalyptus (*Eucalyptus globulus*), guava (*Psidium guajava*) [104], *Abies koreana* [105], Jeju citrus native species: *Citrus obovoides* and *Citrus natsudaidai* [106], *Cryptomeria japonica* [107], citronella grass (*Cymbopogon nardus*) [108], mint (*Mentha spicata*), thyme (*T. vulgaris*), cinnamon (*Cinnamomum zeylanicum*) [109], sweet basil (*Ocimum basilicum*) [110], bitter orange (*Citrus aurantium*), eucalyptus (*Eucalyptus radiata*), juniper (*Juniperus communis*), rose geranium (*Pelargonium asperum*), patchouli (*Pogostemon cablin*), and benzoin (*Styrax benzoe*) [111]. These essential oils can be developed as soaps, creams, gels, etc., to prevent or treat acne, or they can be used in the development of pharmaceutical products such as doxycycline, spironolactone, and minocycline, which are actually used as acne treatments.

Oh et al. [35] reported that the essential oil of Jeju thyme contains 93.5% cymene and has anti-acne effects at the 0.5 mg/mL level against the acne bacteria *P. acnes* and *Propionibacterium granulosum*. However, it is suggested that the efficacy is not very high.

### 5.14. Plant Growth Inhibitory Activity

Kim and Hong [112] found that methanol extract of thyme inhibited the growth of *Echinochloa crus-galli* and *Lemna minor*. Since these results have been studied only in vitro, additional tests should be conducted to determine whether they are effective even under in vivo conditions.

### 5.15. Aromatherapy Activity

Aromatherapy is a technique or practice to improve the psychological and physiological functions of humans using essential oils obtained from various medicinal and aromatic plants [113]. Currently, there are 375~400 types of essential oils used for aromatherapy treatment, and the recommended amount for each oil can be prescribed through an aromatherapist qualified by the National Association for Holistic Aromatherapy. Clary sage, cypress, eucalyptus, geranium, lavender, lemon, and palmarosa are recommended essential oils for beginners [114]. The *Thymus* genus has been widely used for strengthening memory and concentration and calming nerves [12]. Although no studies reporting the aromatherapy effect of thyme essential oil, it is inferred that thyme and island thyme contain almost similar ingredients; thus, a similar effect will be expressed. It is expected that the aromatherapy effect of thyme will greatly help in promoting a comfortable state of mind for those who are tired of fearing the coronavirus that is spreading around the world.

### 5.16. Ground Cover Plant

Thyme has a cover effect due to the fact of its nature of spreading and growing along the ground surface; thus, it is planted under street trees in Jeju Island. Bang et al. [115] experimented on whether thyme was suitable for roof greening, and it was evaluated that the growth condition was good when irrigated for at least 1 week at a soil depth of 5 cm, and it was evaluated as a tree species suitable for low-management roof greening at a soil depth of 10 cm. Youn et al. [116] also argued that thyme is a good rooftop greening plant that can alleviate the urban heat island phenomenon. Thyme can thus winterize, cover the ground, and has excellent dry resistance. Youn et al. [116] also monitored the surface temperature for each rooftop greening planting model and recommended that island thyme is a ground-covered plant that can overwinter and has excellent dry resistance.

**Table 1:** Essential oil isolation from *Thymus quinquecostatus* and its major components.

S. No.	Place of Collection	Extraction Method	Major Components	References
1.	Wolchul, Jiri, and Odae mountains, South Korea	Steam distillation	Odae cultivar—thymol (30.54%), *γ*-terpinene (23.92%), and *p*-cymene (11.13%) Wolchul cultivar—geraniol (42.94%), geranyl acetate (26.49%), and borneol (5.91%) Jiri cultivar—linalool (47.89%), thymol (15.98%), and caryophyllene (7.02%)	[31]
2.	Yantai city, Shandong Province, China	Hydro-distillation	Linalool (52.003%), borneol (10.911%), and anethole (5.325%)	[117]
3.	China	Hydro-distillation	*o*-Cymene, carvacrol, caryophyllene, 2-isopropyl-1-methoxy-4-methylbenzene, and gamma-terpene	[118]
4.	Jeju Island, South Korea	Hydro-distillation	*p*-Cymen-3-ol (50.41%), *p*-cymen-2-ol (24.06%), and cymene (19.04%)	[35]
5.	Jeju high mountain, Jeju middle mountain, Kyeonggi Province, Ulleung Island, and Gangwon Province, South Korea	Thermal desorption gas chromatograph and mass spectrometer	Jeju high mountain—γ-terpinene (18.51%), thymol (13.89%), bicyclo [2.2.1] heptan-2-one (10.61%), and limonene (5.80%) Jeju middle mountain—thymol (35.91%), γ-terpinene (12.13%), and benzene (5.82%) Kyeonggi Province—carvacrol (18.25%), γ-terpinene (8.73%), and thymol (6.69%) Ulleung Island—phenol (13.48%), δ-terpinene (4.21%), and caryophyllene (3.46%) Gangwon Province—carvacrol (19.20%), γ-terpinene (8.83%), and sabinene hydrate (5.55%)	[41]
6.	Gangwon Province, South Korea	Supercritical fluid extraction and water and steam distillation	Supercritical fluid extraction—thymol (77.63%), carvacrol (5.65%), and β-bisabolene (20.65%) Water and steam distillation—thymol (30.44%), β-bisabolene (20.65%), and caryophyllene (6.46%)	[37]
7.	South Korea	Simultaneous and steam distillation extraction	Thymol (39.8%), γ-terpinene (10%), *p*-cymene (9.2%), camphor (5.9%)	[28]
8.	Chungbuk, South Korea	Solid-phase microextraction and simultaneous distillation and extraction	Citral (24.90% and 33.67%), trans-geraniol (36.85% and 39.75%), and geranyl acetate (3.43% and 6.00%)	[33]
9.	Cultivated in Seoul, South Korea	Steam distillation	Thymol (41.7%), γ-terpinene (16%), and *p*-cymene (13%)	[50]
10.	Four regions in China: YL—Shaanxi Province, JB—Shaanxi Province, QY—Gansu Province, and LD—Ningxia Hui Autonomous Region	Hydro-distillation	YL—Shaanxi Province—carvacrol ethyl ether (31.80%), 1,8-cineole (7.23%%), borneol (6.50%), and terpinen-4-ol (4.96%) JB—Shaanxi Province—carvacrol ethyl ether (23.32%), *p*-cymene (19.20%), terpinen-4-ol (10.56%), borneol (5.61%), and 1,8-cineole (5.22%) QY—Gansu Province—*p*-vinyl guaiacol (23.55%), thymol (16.32%), *o*-cymene (12.10%), γ-terpinene (11.11%), and 1,8-cineole (10.16%) LD—Ningxia Hui Autonomous Region—linalool (12.80%) and γ-terpineol (3.04%)	[119]
11.	Laoshan Mountains, Qingdao, China	Steam distillation	Growth period, flowering period, and nearly withered period: linalool—40.31, 39.10, and 45.44%, respectively	[120]

**Table 2:** Biological activities of essential oil, extracts, and components from *Thymus quinquecostatus*.

S. No.	Sample	Biological Activity	Model	References
1.	Essential oil	Antibacterial	*Streptococcus pneumoniae, Staphylococcus aureus, Salmonella enteritidis,* and *Salmonella typhimurium*	[67]
2.	Essential oil	Insecticidal and repellent	*Tribolium castaneum, Lasioderma serricorne,* and *Liposcelis bostrychophila*	[117]
3.	Essential oil	Antimicrobial	*Propionibacterium*	[35]
4.	Essential oil	Antibacterial	*E. coli* 1-deoxy-d-xylulose-5-phosphate reductoisomerase	[73]
5.	Essential oil	Antifungal	Experimental vaginal candidiasis in mice by *Candida albicans*	[61]
6.	Essential oil	Antifungal	*Aspergillus niger, Aspergillus flavus, Candida albicans, Candida utilis, Cryptococcus neoformans, Trichosporon mucoides,* and *Blastoschyzomyces capitatus*	[50]
7.	Essential oil	Antioxidant	DPPH, ABTS, FRAP thiobarbituric acid reactive substances (TBARS) and oxidative stress in zebrafish	[119]
8.	Thymol	Hepatoprotective	Tert-butyl hydroperoxide (t-BHP)-induced oxidative damage in Chang liver cells.	[121]
9.	Thymol (2-isopropyl-5-methylphenol)	Anti-melanogenic	B16F10 cells, inhibitory effect of thymol to tyrosinase, expression level of tyrosinase in B16F10 cells	[90]
10.	Galuteolin	Skin whitening	B16/F10 melanoma cells	[122]
11.	Water extract	Antioxidant	(LPS) To induce inflammation and oxidative stress in RAW 264.7 macrophages; nitric oxide and H_2_O_2_ assay and mitochondrial ATP assay	[1]
12.	Polysaccharides and its fractions	Antioxidant and inhibition of digestive enzymes	DPPH, ABTSagainst 2, 2’-azo-bis-(2-methylpropylimid)-dihydrochloride (AAPH)-induced oxidative stress in a zebrafish model; α-amylase and α-glucosidase	[123]
13.	Water and 70% ethanolic extracts	Antioxidant, cytoprotective, and anti-apoptotic	FRAP, ferric thiocyanate (FTC) and thiobarbituric acid (TBA) methods; t-BHP-induced toxicity	[124]
14.	Extracts obtained by supercritical fluid extraction, simultaneous distillation and extraction, and microwave-assisted extraction	Antioxidant and antimicrobial	*Staphylococcus aureus, Bacillus cereus, Salmonella typhimurium, Bacillus subtilis, Escherichia coli,* and *Saccharomyces cerevisiae*; nitrite scavenging, and DPPH	[36]
15.	50% methanol extract	Alpha-amyalse/-glucosidase inhibition and antioxidant	Alpha-amyalse/-glucosidase ORAC system; maltase and sucrose inhibition	[56]
16.	Extract	Hepatoprotective	Chronic alcohol-induced liver injury in C57 mice	[125]
17.	Ethanol extracts—ethyl acetate fraction	Anti-tumor	Human leukemia cell lines K562 and HL-60	[126]
18.	Methanol extract—the ethyl acetate fraction	Antioxidant, antimicrobial, and antidiabetic	DPPH scavenging and reducing power assays; *Kocuria rhizophila* and *Staphylococcus epidermidis*; α-glucosidase and α-amylase inhibition	[55]
19.	70% Ethanol	Anti-aging effect	Human keratinocytes	[100]
20.	Ethyl acetate Extract 2(S)-5,7,3’,5’-tetrahydroxyflavanone, (+)-taxifolin, (+)-aromadendrin, rosmarinic acid, caffeic acid, protocatechuic acid, and protocatechuic aldehyde	Pancreatic lipase inhibition	Enzyme-based method	[92]
21.	70% Ethanol extract	Antioxidant and antimicrobial	DPPH scavenging activity; *Enterococcus faecalis, Listeria monocytogenes, Citrobacter* *Freundii,* and *Escherichia coli*	[58]
22.	Supercritical fluid extraction	Antimicrobial	Fungus—*Ascosphaera apis*	[64]
23.	Ethanol extract: (1) danshensu, (2) vanillic acid, (3) chlorogenic acid, (4) galuteolin, (5) scutellarin, (6) apigenin	Antioxidant	Response surface methodology based on its DPPH radical scavenging activity	[127]
24.	Polyphenol-rich fraction	Cardioprotective	Myocardial ischemia injury in mice	[128]
25.	High-polar extract (ethanol) and polyphenol-rich fraction (PRF)	Anticerebral ischemia-reperfusion injury effect	Free radicals and zebrafish embryos; transient middle cerebral artery occlusion (tMCAO) model in rats	[129]
26.	Extract	Antifungal	*Cladosporium cucumerinum*	[62]

## 6. Conclusions

Thyme is an aromatic medicinal plant and is widely distributed in Korea. At present, thyme is used only as an ornamental and ground cover plant. There are several kinds of foreign thymes (thyme, *Thymus vulgaris* L.) that are sold as thyme on the market. *T. quinquecostatus* has antioxidant, antimicrobial, anticancer, anti-inflammatory, analgesic, anti-obesity, and anti-acne activities. Various physiological activities, such as sleep prolonging, soothing, whitening, skin protection, skin aging inhibitory, and plant growth inhibitory properties have been reported, but industrial application of *T. quinquecostatus* is insufficient. Thyme is expected to be actively utilized in various industries such as food, pharmaceuticals, pesticides, and aromatherapy; thus, the total area of thyme cultivation should be expanded.
